# Assessing the Risks of Potential Bacterial Pathogens Attaching to Different Microplastics during the Summer–Autumn Period in a Mariculture Cage

**DOI:** 10.3390/microorganisms9091909

**Published:** 2021-09-09

**Authors:** Dandi Hou, Man Hong, Yanting Wang, Pengsheng Dong, Huangwei Cheng, Huizhen Yan, Zhiyuan Yao, Daoji Li, Kai Wang, Demin Zhang

**Affiliations:** 1 State Key Laboratory for Managing Biotic and Chemical Threats to the Quality and Safety of Agro-Products, Ningbo University, Ningbo 315211, China; houdandi@nbu.edu.cn; 2 School of Marine Sciences, Ningbo University, Ningbo 315211, China; hmman_hong@163.com (M.H.); wytsunshine@163.com (Y.W.); dpsh@foxmail.com (P.D.); cheng_huangwei@163.com (H.C.); hzyan1995@163.com (H.Y.); 3 School of Civil and Environmental Engineering, Ningbo University, Ningbo 315211, China; yaozhiyuan@nbu.edu.cn; 4 State Key Laboratory of Estuarine and Coastal Research, East China Normal University, Shanghai 200062, China; daojili@sklec.ecnu.edu.cn; 5 Collaborative Innovation Center for Zhejiang Marine High-Efficiency and Healthy Aquaculture, Ningbo 315211, China

**Keywords:** microplastic pollution, pathogenic microorganism, aquaculture, risk assessment, pathogen database, aquatic ecosystem

## Abstract

As microplastic pollution continues to increase, an emerging threat is the potential for microplastics to act as novel substrates and/or carriers for pathogens. This is of particular concern for aquatic product safety given the growing evidence of microplastic ingestion by aquaculture species. However, the potential risks of pathogens associated with microplastics in mariculture remain poorly understood. Here, an in situ incubation experiment involving three typical microplastics including polyethylene terephthalate (PET), polyethylene (PE), and polypropylene (PP) was conducted during the summer–autumn period in a mariculture cage. The identification of potential pathogens based on the 16S rRNA gene amplicon sequencing and a custom-made database for pathogenic bacteria involved in aquatic environments, was performed to assess the risks of different microplastics attaching potential pathogens. The enrichment of pathogens was not observed in microplastic-associated communities when compared with free-living and particle-attached communities in surrounding seawater. Despite the lower relative abundance, pathogens showed different preferences for three microplastic substrates, of which PET was the most favored by pathogens, especially potentially pathogenic members of *Vibrio*, *Tenacibaculum*, and *Escherichia*. Moreover, the colonization of these pathogens on microplastics was strongly affected by environmental factors (e.g., temperature, nitrite). Our results provide insights into the ecological risks of microplastics in mariculture industry.

## 1. Introduction

Microplastics have emerged as a major source of anthropogenic pollution in marine environments [1,2]. This contamination is now widespread, recalcitrant, and likely to continue unabated into the future [3]. Numerous studies have revealed that microplastics cause adverse effects on marine organisms [4,5,6] and even humans [7] through direct physical damage [8] and indirect toxicities caused by adsorbed pollutants [9] or plastic additives [10]. Besides that, the recent realization that this novel substrate in the marine environment may colonized by microorganisms that include pathogens has intensified interest in the microbial ecology of this biotope and its potential impact on aquatic ecosystems [2,3].

Their hydrophobic hard surfaces, novel polymer carbon sources and long-distance transport make microplastics a unique substrate for microbial colonization within marine environments [11]. Once they enter aquatic environments, a biofilm will quickly develop on the microplastic surfaces [12]. Zettler et al. first described the ‘plastisphere’ that is taxonomically distinct from the surrounding water and proposed that some plastisphere members may be opportunistic pathogens, such as *Vibrio* members that dominated a polypropylene sample collected from the North Atlantic [13]. The presence of numerous pathogens (for example, members of the *Vibrio parahaemolyticus*, *Escherichia coli* or *Arcobacter* spp.) on microplastic surfaces from across oceanic regions has since been widely demonstrated [14,15,16]. This raises the important question as to whether the increasing amount of microplastics in global oceans provides greater opportunities for pathogens to be colonized, transported and transmitted to potential hosts, compared to the opportunities provided by other, natural particles.

Over the past decade, research effort has been undertaken on exploring the pathogen risk of microplastics in various aquatic ecosystems, such as coastal water [17], bays [18], estuaries [19], urban rivers [20]. Some studies have suggested that microplastics can serve as vectors to increase the survival of potential pathogens, and transfer pathogens to marine organisms or formerly unaffected ecosystems [21]. Members of the genus *Vibrio* were found to be particularly enriched on microplastics in the Bay of Brest [18] and the North and Baltic Seas [14]. Wu et al. reported the selective enrichment of pathogenic members of *Pseudomonas* on microplastics in a river [22]. In several pathogen-rich environments such as sewage [23] and landfill leachate [24], microplastics have also been observed to selectively enrich pathogens from the surrounding environments. However, other studies have disputed the preferential colonization of microplastics by pathogens [25,26]. The divergence of these results may be due to the huge variety in the biochemical composition of different plastic polymers, as well as the environmental conditions and pathogens richness of different aquatic ecosystems. Moreover, studies to date that have discovered pathogenic species have generally looked at one or a few specific pathotypes only and not the whole microbial community; this does not allow for complete comparisons when looking at the context of the plastisphere as a whole [3]. It is necessary to study in depth the pathogen colonization on different types of microplastics exposed to different pathogen-rich environments in the context of the overall community in terms of risk assessment.

With the growth of the global human population and the over-exploitation of wild stocks, aquaculture is urgently needed to develop rapidly to meet the large demand of seafood production and future sustainable development of fisheries [27]. The outbreak of microbial diseases is one of the biggest issues faced by the aquaculture industry, hence any factors that may increase disease are a particular concern [3]. Aquaculture facilities use large amounts of plastics for floats, nets, pens, and lines, among other equipment [28]. As an important contributor to marine microplastics, these facilities might increase the chance that potential pathogens could colonize microplastic surfaces [29,30]. Moreover, there is a growing body of evidence that commercial seafood and aquaculture species absorb microplastics [31]. Despite the potential threats to aquatic biosecurity and seafood safety, little is known about the pathogen risks from microplastics in mariculture.

Here, an in situ incubation experiment involving three typical categories of microplastics was conducted during the summer–autumn period, when there is a high risk of disease outbreak, in a nearshore mariculture cage. Our work goes beyond the general characteristics of bacterial community attaching onto the microplastics, focusing on the attachment risks of potential pathogens on different microplastics in the typical fishery environment with extensive microplastic pollutions and pathogenic risks. Moreover, a custom-made database for pathogenic bacteria involved in aquatic environments was applied, which would provide an effective way to identify potential pathogens in the context of the whole community based on 16S rRNA gene amplicon sequencing. Specifically, we aim to address three questions: (1) whether microplastics exposed to mariculture pose a risk of pathogen enrichment; (2) does the colonization of pathogens on microplastics vary with different polymer types; and (3) which environmental factors in mariculture may be related to the colonization of pathogens on microplastics?

## 2. Materials and Methods

### 2.1. Experimental Design and Data Collection

An in situ incubation experiment was performed in a large yellow croaker (*Pseudosciaena crocea*) cage in Xiangshan Harbor (121°45′25′′ E, 29°32′2′′ N) in the East China Sea. The microplastic preparation, experimental design, sample collection, 16S rRNA gene sequencing, and sequence processing were described in our previous study [32]. Briefly, three types of microplastic particles (polyethylene terephthalate, PET; high-density polyethylene, PE; expanded polypropylene, PP) were prepared by fragmenting, sieving, ultrasonic cleaning, and sterilizing the three typical plastic materials (PET bottles, PE film, and PP cushioning foam) as previously described [32]. The three prepared microplastics (200 particles of each substrate) were respectively placed in three porous hollow balls (12 cm in diameter) wrapped with 60-mesh nylon nets. The three balls containing three different microplastics were bundled into one group. Twenty groups of microplastic particles were hung in five positions (as five biological replicates, four corners and the midpoint of one side) of the squared cage and fixed at a water depth of 0.5 m (Figure S1). They were incubated for three months (August to October) during the summer–autumn period, when the temperature and the risk of disease outbreak were generally highest across the entire culture period. One of four groups of microplastic particles at each position was collected at days 15 (9 August 2017), 30 (23 August), 60 (24 September), and 90 (24 October). Five replicate seawater samples were also collected in situ at five microplastic placement points at each sampling day. The seawater environmental factors were monitored using standard methods [33] and the metadata of the environmental factors are provided in Dataset S1. Additionally, approximately 1 L of seawater was filtered sequentially through 20-μm, 2-μm, and 0.2-μm polycarbonate membranes (Millipore, Boston, MA, USA) to collect large particle-attached (LPA, >20 μm), small particle-attached (SPA, 2–20 μm), and free-living (FL, 0.2–2 μm) planktonic prokaryotes, respectively [34,35,36,37]. Total DNA on the surface of microplastics (0.5 g) and water fraction filters was extracted using a PowerSoil^®^ DNA Isolation Kit (MOBIO, Carlsbad, CA, USA). The V4–V5 region of the 16S rRNA genes was amplified using the dual-indexed bacterial–archaeal universal primer set 515FY-926R [38,39] and sequenced as previously described [32]. The sequence data are available under accession number DRA010047 in the Sequence Read Archive of DDBJ (https://ddbj.nig.ac.jp/DRASearch, accessed on 3 September 2021). The sequences were processed using QIIME v1.9.1 [40] as previously described [32].

### 2.2. Construction of Bacterial Pathogens Database

A database of bacterial pathogens in aquatic environments [41] constructed by collecting the pathogenic species/strains information and the corresponding 16S rRNA gene sequences was applied in this study. Six major categories of bacterial microorganisms that are potentially pathogenic to fish, human, mammal, invertebrate, plant, and multi-hosts (cross-host) were included in this database. The list of fish pathogens and related information were mainly collected from the reports of Austin et al. [42] and Fang et al. [43]. Human pathogens (mainly fecal–oral transmission) were derived from the Virulence Factor Database (VFDB, http://www.mgc.ac.cn/VFs/, accessed on 1 March 2021) [44] and a bacterial pathogens database constructed by Chen et al. [45]. The information of other hosts and cross-host pathogens were collected from the KEGG database of Antimicrobial Resistance [46]. The taxonomy information of collected pathogens was calibrated by the database of the List of Prokaryotic names with Standing in Nomenclature (LPSN) [47,48]. The 16S rRNA gene sequences of species or strains in pathogens list were extracted from the NCBI nucleotide database (http://www.ncbi.nlm.nih.gov/, accessed on 1 March 2021). If the pathogenic strains of a pathogenic species were undefined in references, the 16S rRNA gene sequence of the type strain of this species was used as the reference sequence in the database. The database currently features information on 9070 pathogens and corresponding 16S rRNA gene sequence data, including 14 phyla, 116 families, 221 genera, and 1097 species (Table S1).

### 2.3. Taxonomic Assignment of Bacterial Pathogens

After sequence processing using QIIME, a prokaryotic OTU table was generated and rarefied at 25,200 reads per sample (corresponding to the smallest sequencing depth for any of the samples). To determine the relative abundance of potential pathogens on/in microplastics and water fractions, the representative sequence of each OTU was aligned with the 16S rRNA full-length gene sequences in the constructed database using the USEARCH (v11.0.667) global alignment algorithm (default settings). The hit outputs were further filtered using the strict criteria of E-value < 1 × 10^−6^ and sequence identity > 99% to taxonomically annotate the pathogenic bacteria and generate the potentially pathogenic OTU table.

### 2.4. Statistical Analyses

Nonmetric multidimensional scaling (NMDS) analysis based on the Bray–Curtis dissimilarity was performed to visualize the variations of pathogenic communities among different sampling days and substrates using the QIIME script *nmds.py*. Analysis of similarity (ANOSIM) and permutational multivariate analysis of variance (PERMANOVA) based on Bray–Curtis dissimilarity were conducted using the ‘anosim’ and ‘adonis’ functions of the R package ‘vegan’ [49], respectively, to test the significance of compositional deference of pathogenic communities between sampling days or between substrates. Mantel tests with 999 permutations were used to test correlations between seawater environmental factors (Euclidean distance) and β-diversities (Bray–Curtis distance) of pathogenic community. Heatmaps showing relative abundances of dominant pathogenic OTUs (top 30 OTUs in relative abundance) and their correlations with environmental factors, were created by the R package ‘pheatmap’ [50]. A maximum-likelihood phylogenetic tree was constructed using MEGA 7 to present phylogenetic relationships among these dominant OTUs [51]. Ternary plot was constructed to identify the representative pathogenic OTUs on the surfaces of PET, PE, and PP using the R package ‘ggtern’ [52].

## 3. Results

### 3.1. Overview of Potential Bacterial Pathogens

A total of 134 OTUs representing potentially pathogenic bacteria were obtained through sequence alignment with the bacterial pathogens database in aquatic environments, including 72 fish pathogenic OTUs (53.73%), 29 human pathogenic OTUs (21.64%), 3 plant pathogenic OTUs (2.24%), 2 mammal pathogenic OTUs (1.49%), and 28 cross-host pathogenic OTUs (20.90%, Figure S2A). Most pathogenic OTUs (65.67%) co-occurred in water fractions and microplastics, while only 25% and 4% of pathogenic OTUs were unique to water fractions and microplastics, respectively (Figure S2B).

### 3.2. Distribution of Pathogens across Different Microplastics and Water Fractions

**Figure 1:**
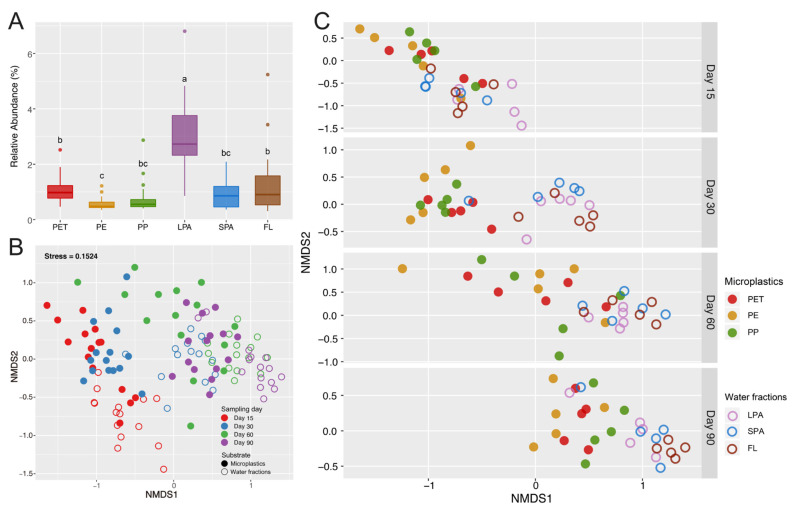
Relative abundance and community succession of potential pathogens on/in microplastics and water fractions. (**A**) Relative abundance of potential pathogens in prokaryotic communities of microplastics and waters. The different lowercase letters indicate significant difference among various substrates (*p* < 0.05). PET: Polyethylene terephthalate; PE: Polyethylene; PP: Polypropylene; LPA: Large particle-attached; SPA: Small particle-attached; FL: Free-living. (**B**) Non-metric multidimensional scaling (NMDS) ordination plot based on the Bray–Curtis dissimilarity showing the temporal variation of pathogenic community structure on/in microplastics and water fractions. (**C**) Differences in the pathogenic community structures on/in different microplastic substrates and water fractions at each sampling day.

**Figure 2:**
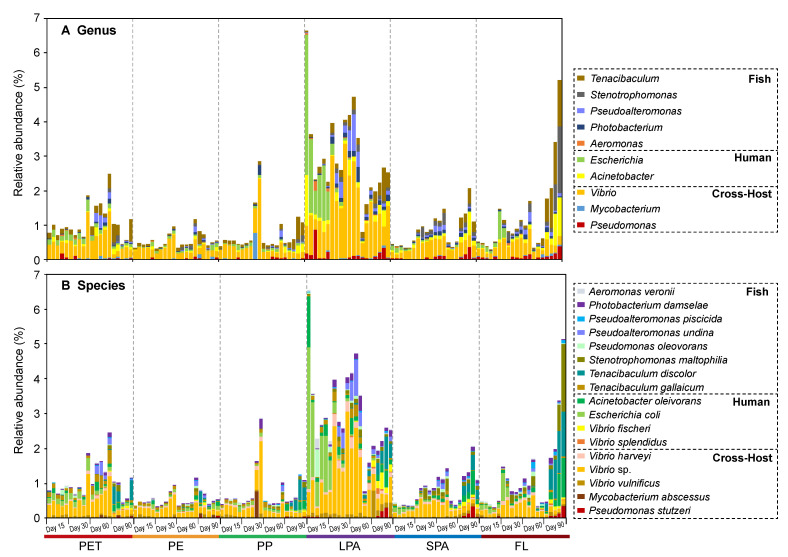
Compositions of the dominant pathogenic bacterial genera ((**A**) relative abundance > 0.1% in at least one sample) and the dominant pathogenic bacterial species (**B**) on/in microplastics and water fractions.

The relative abundance of pathogens on LPA (3.09%) was significantly higher than that on microplastics (0.81%) and on/in other water fractions (SPA, 0.87%; FL, 1.29%; Figure 1A). Among the three types of microplastics, PET had the highest abundance of pathogens (1.10%). The pathogenic abundance on PE (0.57%) was significantly lower than all other substrates including microplastics and water fractions. The pathogens on/in microplastics and water fractions were mainly affiliated with the genera *Vibrio* (0.72%), *Escherichia* (0.23%), *Tenacibaculum* (0.20%), and *Acinetobacter* (0.16%, Figure 2A). *Vibrio* sp. (0.42%), *Escherichia coli* (0.16%), *Tenacibaculum discolor* (0.10%), and *Acinetobacter oleivorans* (0.10%) were the dominant pathogenic species of these genera (Figure 2B).

### 3.3. The Succession of Pathogens on/in Microplastics and Water Fractions

In general, pathogenic community structures of microplastics and water fractions were both clustered with sampling day (Figure 1B), indicating clear succession of pathogenic community over time. PERMANOVA results corroborated that sampling day comprised the largest source of pathogenic community variation (23.34%, *p* < 0.001; Table S2). The pathogenic communities in water fractions showed more visible succession patterns than those on microplastics (Figure 1B) as statistically indicated by R_ANOSIM_ values (Table S3). The pathogenic community compositions between microplastics and water fractions were significantly different at each sampling day (Figure 1C) (all *p* < 0.001, Table S4).

### 3.4. Representative Pathogens on Three Microplastics

**Figure 3:**
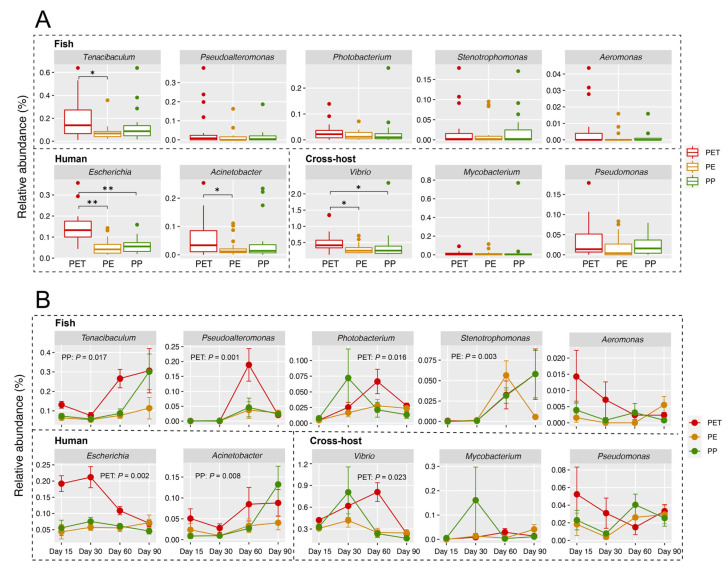
Relative abundance and dynamics of dominant pathogenic genera (relative abundance > 0.1% in at least one sample) on microplastics. (**A**) Differences in the relative abundance of the dominant pathogenic genera on the three microplastics (multiple comparisons after Kruskal–Wallis test, * *p* < 0.05, ** *p* < 0.01). (**B**) Dynamic changes in the relative abundance of dominant pathogenic genera on different microplastics with the sampling day. Microplastics with significant differences (*p* < 0.05) in the relative abundance of dominant pathogenic genera among different sampling days were marked in the figures.

A total of ten dominant pathogenic genera (relative abundance > 0.1% in at least one sample) were detected on the microplastics in this study (Figure 2 and Figure 3). Among them, the relative abundances of genus *Tenacibaculum* and *Acinetobacter* on PET were significantly higher than those on PE (*p* < 0.05). *Escherichia* (*p* < 0.01) and *Vibrio* (*p* < 0.05) on PET were significantly more abundant than those on both PE and PP (Figure 3A). Across sampling days, the relative abundances of these dominant pathogenic genera (except for *Stenotrophomonas*, *p* = 0.003) on PE had no significant fluctuations (Figure 3B). In contrast, the other two microplastics showed temporal variations in the abundance of pathogenic genera. For example, the abundance of *Tenacibaculum* (*p* = 0.017) and *Acinetobacter* (*p* = 0.008) on PP had a significant increasing trend over time. On PET, the abundance of *Pseudoalteromonas*, *Photobacterium*, and *Vibrio* showed a similar trend, with a peak at day 60, while the abundance of *Escherichia* sharply decreased from day 30 (*p* = 0.002, Figure 3B).

**Figure 4:**
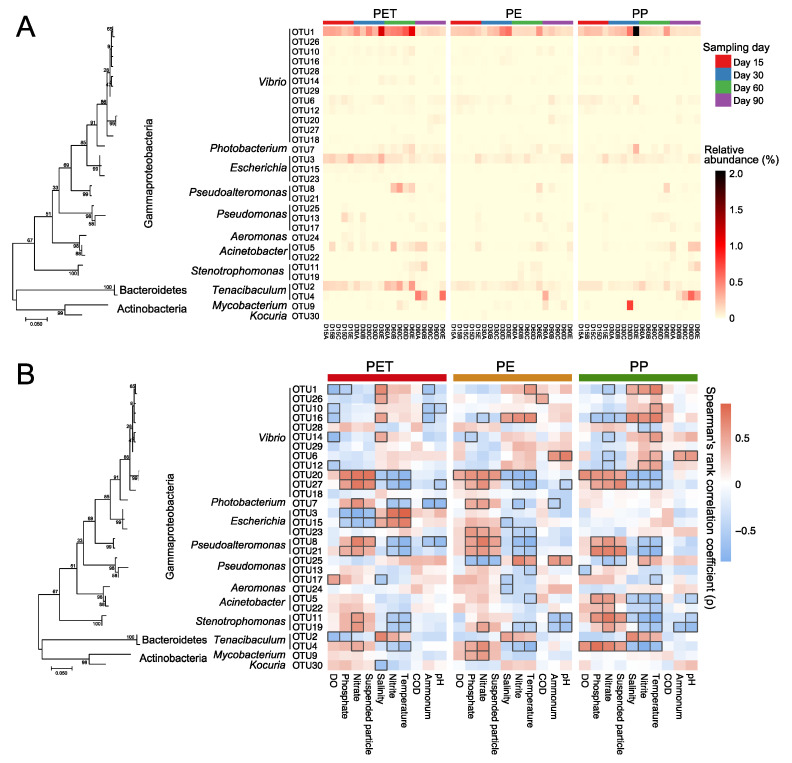
The relative abundances of dominant pathogenic OTUs (top 30 OTUs in relative abundance) and their correlations with seawater environmental factors. (**A**) Relative abundances of dominant pathogenic OTUs of microplastics at different sampling days. The phylogenetic tree was constructed using the maximum likelihood method in MEGA 7. (**B**) Spearman’s rank correlations between the relative abundances of dominant pathogenic OTUs on microplastics and environmental factors of seawater. Significant Spearman’s correlations (*p* < 0.05) were noted with black frames. DO, dissolved oxygen; COD, chemical oxygen demand.

**Figure 5:**
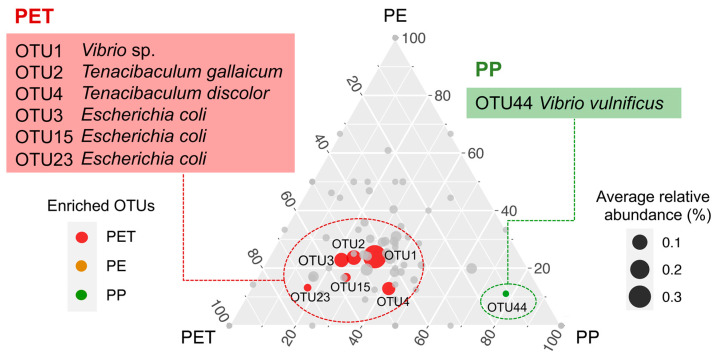
Ternary plot illustrating the representative/enriched pathogenic OTUs of microplastics. Each circle represents one OTU. The size of each circle represents its relative abundance. The position of each circle is determined by the contribution of the indicated microplastic substrate to the total relative abundance. Red circles and green circles mark OTUs specifically enriched on PET and PP, respectively.

At the OTU level, 86.7% of the top 30 pathogenic OTUs in relative abundance on the three microplastics belong to Gammaproteobacteria, including *Vibrio* (40%), *Escherichia* (10%), *Pseudomonas* (10%), and others (Figure 4A). *Vibrio* OTU1 (*Vibrio* sp.) had an overwhelming advantage on the surfaces of the three microplastics, but its abundance greatly decreased at day 90. *Tenacibaculum* OTU2 (*Tenacibaculum gallaicum*), as the second most abundant pathogenic OTU, likewise experienced a sharp decline at day 90. Instead, OTU4 (*Tenacibaculum discolor*) became the dominant pathogenic OTU (especially on PET and PP). That is, species turnover occurred within the genus *Tenacibaculum* as the incubation of the microplastics went on. The ternary plot showed the representative pathogenic OTUs on each type of microplastics (Figure 5). Compared with PP and PE, six potential pathogenic OTUs were enriched on PET, including *Vibrio* OTU1 (*Vibrio* sp.), *Tenacibaculum* OTU2 (*Tenacibaculum gallaicum*) and OTU4 (*Tenacibaculum discolor*), and *Escherichia* OTU3, OTU15, and OTU23 (*Escherichia coli*). Only one potential pathogenic OTU (OTU44, *Vibrio vulnificus*) was enriched on PP, while no enriched pathogenic OTU was found on PE.

### 3.5. Correlation between the Microplastic-Attached Pathogens and Environmental Factors

In comparison with microplastics, the pathogenic community structures in water fractions showed stronger correlations with environmental factors based on Mantel tests (Table 1). LPA and SPA were significantly correlated with all environmental factors except chemical oxygen demand (COD). For the three types of microplastics, PET showed the strongest correlation with most environmental factors (including temperature, nitrate, dissolved oxygen (DO), salinity, phosphate, and nitrite, all *p* < 0.05), followed by PP. Among all environmental factors, temperature, nitrate and DO were the most relevant to the pathogenic community structures on the three microplastics.

Spearman’s rank correlation analysis also showed that temperature was an important factor affecting the colonization of the dominant pathogen OTUs (top 30 OTUs in relative abundance) on the three microplastics (Figure 4B). In addition, nutrient-related factors also exhibited a strong correlation with some pathogenic OTUs. For example, the relative abundances of *Vibrio* OTU20 (*Vibrio fischeri*), OTU27(*Vibrio splendidus*), and *Pseudoalteromonas* OTU21 (*Pseudoalteromonas piscicida*) on three microplastics were all significantly correlated with nitrate, nitrite, and phosphate. Most members of the three most abundant pathogenic genera (*Vibrio*, *Tenacibaculum*, and *Escherichia*) on microplastics were generally positively correlated with temperature, nitrite, and salinity.

## 4. Discussion

### 4.1. Pathogenic Bacteria Were Not Be Enriched on the Microplastics Compared with the Surrounding Environments

Organic aggregate particles in water are islands for microbial assemblages and sometimes for pathogens [53]. The pathogens could attach to particles and might further mature into surface biofilms, thereby gaining considerable survival advantages such as abundant nutrient assimilation and increased resistance to adverse conditions [54,55]. Thus, particles could be a critical factor for the fate and survival of pathogens in water [56]. In this study, the highest abundance of pathogens was found to be attached to large particles suspended in the water of the mariculture cage, suggesting that pathogens are more likely to prefer particle-attached lifestyle rather than free-living way in the water. Similarly, as ubiquitous particles in seawater, microplastics represent an important environmental substrate for the colonization of microbes from the surrounding water column [3]. Our previous report showed that the microplastic-associated prokaryotic communities (both α- and β-diversity) were significantly distinct from free-living (FL) and small particle-attached communities (SPA) in the surrounding water but highly similar to the large particle-attached communities (LPA) [32]. In the present study, however, the relative abundances of potential pathogens found on microplastics were comparable with those present in SPA and FL, and were far from those in LPA, indicating that microplastics did not enrich the pathogens compared with the surrounding environments in mariculture from the perspective of relative abundance of pathogenic bacteria. Some other studies have indeed observed the selective enrichment of several potential pathogenic species by microplastics [14,18,22], but one recent meta-analysis across the North Sea, the Baltic Sea, and the Yangtze Estuary supported our result, which concluded that the median relative abundances of a variety of potential pathogens found on microplastics remained below or similar to those from communities associated with natural control surfaces (wood, cellulose, or glass) and the particle-attached water fractions [57]. All the above evidence revealed that microplastics do not per se represent a higher risk of enriching marine pathogenic microorganisms when compared with natural particles.

Although the pathogens on the microplastics appeared in lower relative abundances, the pathogenic community succession in water fractions in this study was more visible than that on microplastics over sampling time, illustrating that the pathogens colonized on microplastics may maintain a more stable community than those free-living or attached to water particles. Combined with the longer residence times and transportability, microplastics may enable related pathogens to travel longer distances horizontally and vertically in the ocean, compared to many non-floating particles or particles that degraded in a shorter time. Therefore, microplastics might not be the only or preferred habitat of potential pathogens, but over a long term, it may become an important transmission vector as the abundance of microplastics in the environment increases [58]. In particular, the frequent use of plastic infrastructure in aquaculture operations could further pose biosecurity and human health risks [28]. However, it is important to note that it still does not suggest that microplastics act as specific vectors for pathogen dispersal [59] and this evidence from molecular sequence data does not prove its pathogenicity or toxicity [2], but warrants further study.

### 4.2. The Colonization and Succession of Pathogens on Microplastics Varied with Different Substrates

In general, the exposure/sampling time, geographical location and surrounding environmental conditions are considered to be the main drivers of biofilm formation on microplastic surface, with polymer type thought to play a less significant role [3,26,58,60,61]. As expected, sampling day in this study overwhelmed polymer type as the major determinant on the community variation. Nonetheless, different types of microplastic polymers have clearly differentiated in terms of abundance, composition, and succession of potential pathogenic community. Among the three microplastic substrates, PET was more favored by pathogens, followed by PP. Our previous research has demonstrated that PET had a stronger substrate specificity than PE and PP to assemble its distinct “plastisphere” by a stronger selection or active dispersal of species from the water microbial pool [32]. These results may be due to the structure, surface charge, manufacturing protocol, lability of microplastics, or some combination of variables [61]. During the 90-day incubation, the pathogens on the PE surfaces maintained a low abundance. Hence, considering only the colonization abundance of pathogens, the risk caused by PE would be less than the other two, especially PET. Certainly, it is necessary to incorporate some other factors into our further comprehensive evaluation of pathogenic risks, such as the dispersal and toxicity of pathogens, as well as the possibility of microplastic ingestion and disease occurrence [2,3].

Members of genera *Vibrio*, *Tenacibaculum*, and *Escherichia* were detected as the dominant potential pathogens attached to the three microplastics in this study, which were particularly abundant on PET. Among them, *Vibrio* appeared with the highest proportion (40%). It is well-known that *Vibrio* is a ubiquitous bacterial genus with wide-ranging and variable habitat preferences, encompassing free-living, host-associated and biofilm-associated representatives [61,62]. Members of the genus detected on the surfaces of microplastics here, including *Vibrio harveyi*, *Vibrio vulnificus*, *Vibrio fischeri*, *Vibrio splendidus*, and some other *Vibrio* spp., are human and animal pathogens that have caused major pandemics and countless epidemics across the globe [61], as well as being the most common pathogens of fish and shellfish that have inflicted expensive losses on aquaculture enterprises [63]. Many opportunistic pathogens for fish species are also included in the genus *Tenacibaculum* [64], such as *Tenacibaculum discolor* and *Tenacibaculum gallaicum* in the present work, which were observed as potentially pathogenic colonizers of microplastics with a clear preference for polymer types. These two species, isolated from sole (*Solea senegalensis*) and turbot (*Psetta maxima*) culture systems [65], have been identified as the causal agent of tenacibaculosis in fish [64]. It is noticed that the two species showed completely opposite dynamic trends in relative abundance throughout the incubation experiment. Although it has not been further explored, these two *Tenacibaculum* species may have differing environmental requirements and tolerances, and thus local environmental changes can modify the ratios of these two pathogens on microplastics. In addition, microplastics (especially PET) also contained human pathogenic and multidrug-resistant *Escherichia coli* [19]. Studies have demonstrated that these pathogens may form cohesive groups within which they easily exchange genetic elements to confer greater antibiotic resistance, as well as regulate virulence [19,61,66]. Therefore, the concern about microplastics serving as vectors of pathogens may well be compounded by the potential for dissemination of antibiotic-resistance genes associated with the plastisphere.

### 4.3. High Temperature and Nitrite in Mariculture May Increase the Risk of Pathogen Attachment on Microplastics

Microplastic-associated pathogenic populations in general, and potentially dominant pathogenic species (such as members of *Vibrio*, *Escherichia*, *Tenacibaculum*) in particular, were strongly affected by environmental conditions of the surrounding water, mostly temperature and nutrient. Taking *Vibrio* as an example, we observed high occurrence of potentially pathogenic species of this taxon in plastisphere, particularly in the summer months (August and September), which may be due to the bloom of *Vibrio* in aquaculture water in response to higher water temperatures. Some researchers identified seawater temperature as the major factor influencing the occurrence and infection rate of pathogenic *Vibrio* [67,68]. For instance, Sobrinho et al. have confirmed that *Vibrio parahaemolyticus* favors warmer water temperatures [69]. And it is well documented that rising seawater temperatures in the North Sea over the past 45 years have been correlated with higher numbers of *Vibrio* species and infections from bathing in the ocean [70]. While temperature is the major factor structuring the occurrence of pathogens on microplastics and its surrounding water, the high nutrient level caused by the accumulation of residual feed and excrement is another inescapable pathogen-associated factor in mariculture environments, when compared with other aquatic ecosystems. Higher nutrients could improve the survival of pathogens due to reduced competition for nutrients and lead to more rapid reproductions of the pathogenic community [17,26]. Additionally, higher nutrients could lead to a more rapid establishment of, and perhaps more consolidated, pathogenic population on microplastics [17]. In summer, high temperature, low oxygen, and excessive nitrite usually co-occur as thorny issues in mariculture [71]. In this study, we did observe lower dissolved oxygen and higher nitrite in mariculture water during high temperature period, which exhibited strong correlations with more colonization of pathogens on microplastics. Excessive nitrite exposure may induce immune suppression in fish, thereby providing more opportunities for pathogen infection [72]. The different seasons covering a broader range of temperature, as well as diverse environmental conditions, should be considered in further work to better understand the relationships between microplastics and microorganisms in aquatic ecosystems, and the factors that promote the colonization of pathogens on microplastics.

## 5. Conclusions

In this study, the potential pathogens in prokaryotic communities associated with different microplastics and water fractions in a mariculture cage were assessed by sequence alignment with a custom-made database of bacterial pathogens. In terms of the overall abundances of potential pathogen communities, PET was most favored by pathogens among the three microplastic substrates. However, microplastics including PET did not show a higher risk of enriching pathogens when compared with water fractions. Nevertheless, some properties of microplastics (e.g., long-distance transport, long-term retention) that are distinct from natural particles may increase the potential risks of microplastics as pathogen vectors to biosecurity and human health. Additionally, environmental conditions also affect the colonization of pathogens on microplastics. In a mariculture environment where both microplastics and pathogens are abundant, high temperature and nitrite may increase the risk of pathogen attachment on microplastics. Although this study provides an effective way to identify pathogens based on a pathogens database, it still has the following limitations. The database cannot fully cover all potential pathogens involved in aquatic environments, which may inevitably underestimate the overall abundance of pathogens in this study. Additionally, the relatively short sequence length in this study can only provide a taxonomic resolution at genus/species level. We suggest that a new methodology such as full-length sequencing or a metagenomic approach could provide a broad and accurate profile of microbial communities and should be used in pathogen identification in the future studies. Moreover, future efforts are needed to better understand the transmissibility and pathogenicity of these potential pathogens attached to microplastics, thus providing a comprehensive assessment for the ecological risks of microplastics in the marine environment.

## Figures and Tables

**Table 1:** Pearson’s correlation coefficients (*r*) between seawater environmental factors (Euclidean distance) and β-diversities (Bray–Curtis dissimilarity) of potentially pathogenic bacterial community of microplastics and water fractions.

Environmental Factors	PET	PE	PP	LPA	SPA	FL
Temperature	**0.681**	**0.437**	**0.526**	**0.436**	**0.570**	**0.714**
Nitrate	**0.647**	**0.443**	**0.489**	**0.491**	**0.619**	**0.715**
DO	**0.639**	**0.323**	**0.437**	**0.292**	**0.440**	**0.624**
Phosphate	**0.359**	**0.211**	**0.254**	**0.601**	**0.622**	**0.614**
Salinity	**0.556**	0.179	**0.315**	**0.350**	**0.417**	**0.603**
Nitrite	**0.184**	0.079	0.125	**0.561**	**0.557**	**0.451**
pH	0.026	0.098	−0.110	**0.203**	**0.184**	0.101
Ammonium	−0.026	0.017	−0.062	**0.299**	**0.224**	0.096
Suspended particle	−0.043	−0.104	−0.020	**0.340**	**0.238**	**0.215**
COD	−0.085	−0.108	−0.130	−0.032	0.029	−0.064

*r*: correlation coefficients between pairwise distances of water environmental factors and pathogenic community distances derived from Mantel testes with 999 permutations. Data in bold indicate significant correlations (*p* < 0.05).

## Data Availability

The sequence data are publicly available via the Sequence Read Archive of DDBJ (https://ddbj.nig.ac.jp/DRASearch, accessed on 3 September 2021) under accession number DRA010047.

## Supplementary Materials

The following are available online at https://www.mdpi.com/article/10.3390/microorganisms9091909/s1, Figure S1: A diagram of in situ incubation experiment set-up, Figure S2: Overview of potential bacterial pathogens recovered from microplastics and water fractions, Table S1: Summary of pathogens information in database of bacterial pathogens in aquatic environment, Table S2: Permutational multivariate analysis of variance (PERMANOVA) results testing the effect of sampling day and substrate on composition of pathogenic community based on Bray–Curtis dissimilarity, Table S3: Analysis of similarity (ANOSIM) based on Bray–Curtis dissimilarity for comparison of pathogenic community compositions of microplastics or water fractions between different sampling days, Table S4: Analysis of similarity (ANOSIM) and permutational multivariate analysis of variance (PERMANOVA) based on Bray–Curtis dissimilarity for comparison of pathogenic bacterial community composition between microplastics and water fractions at each sampling day, Dataset S1: The variations of seawater environmental factors on different sampling days.
