# Supplementary material for: Assessing the Risks of Potential Bacterial Pathogens Attaching to Different Microplastics during the Summer–Autumn Period in a Mariculture Cage
Source: Microorganisms. 2021 Sep 9;9(9):1909. doi: 10.3390/microorganisms9091909 (PMC8469625; doi:10.3390/microorganisms9091909)
Supplement: Supplementary file 1 [file microorganisms-09-01909-s001.zip › Supplementary Materials.pdf]

# Supplementary Materials

## Assessing the Risks of Potential Bacterial Pathogens Attaching to Different Microplastics during the Summer–Autumn Period in a Mariculture Cage

Dandi Hou [1,2], Man Hong [2], Yanting Wang [2], Pengsheng Dong [2], Huangwei Cheng [2], Huizhen Yan [2], Zhiyuan Yao [3], Daoji Li [4], Kai Wang [1,2,5*], Demin Zhang [1,2,5*]

**Author institutional addresses:**

[1] State Key Laboratory for Managing Biotic and Chemical Threats to the Quality and Safety of Agro-products, Ningbo University, Ningbo, 315211, China

[2] School of Marine Sciences, Ningbo University, Ningbo, 315211, China

[3] School of Civil and Environmental Engineering, Ningbo University, Ningbo, 315211, China

[4] State Key Laboratory of Estuarine and Coastal Research, East China Normal University, Shanghai, 200062, China

[5] Collaborative Innovation Center for Zhejiang Marine High-efficiency and Healthy Aquaculture, Ningbo, 315211, China

[*] For correspondence. E-mail Kai Wang (wangkai@nbu.edu.cn) or Demin Zhang (zhangdemin@nbu.edu.cn); Tel. 86-574-87600551; Fax 86-574-87608347.

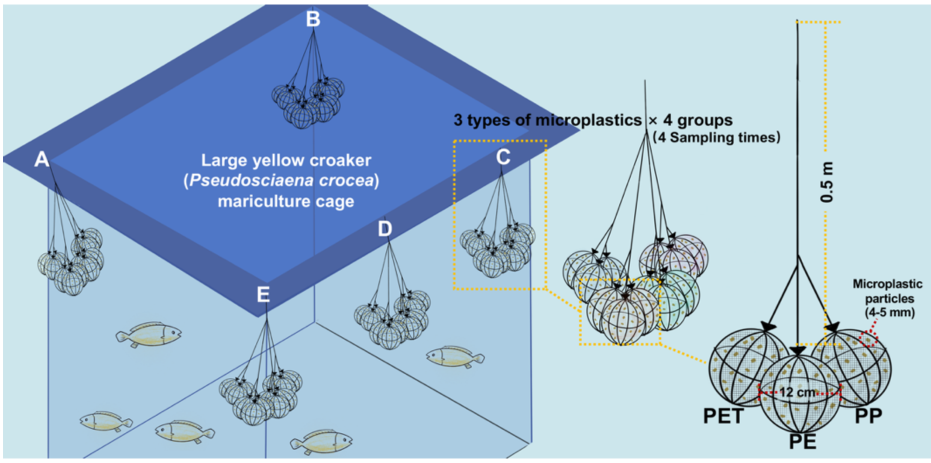


**Figure S1.** A diagram of *in situ* incubation experiment set-up. Porous hollow balls (12 cm in diameter) wrapped with 60-mesh nylon nets, which prevent microplastic particles from falling out of the porous balls and ensure adequate water exchange, were employed to expose three microplastics (polyethylene terephthalate, PET; high-density polyethylene, PE; expanded polypropylene, PP) in a large yellow croaker (*Pseudosciaena crocea*) mariculture cage. Sixty balls (3 types of microplastics × 4 sampling times × 5 replicates) were evenly hung in five positions of the squared cage as five biological replicates and fixed at a water depth of 0.5 m. Three balls containing three microplastics were recovered from each position at each sampling day.

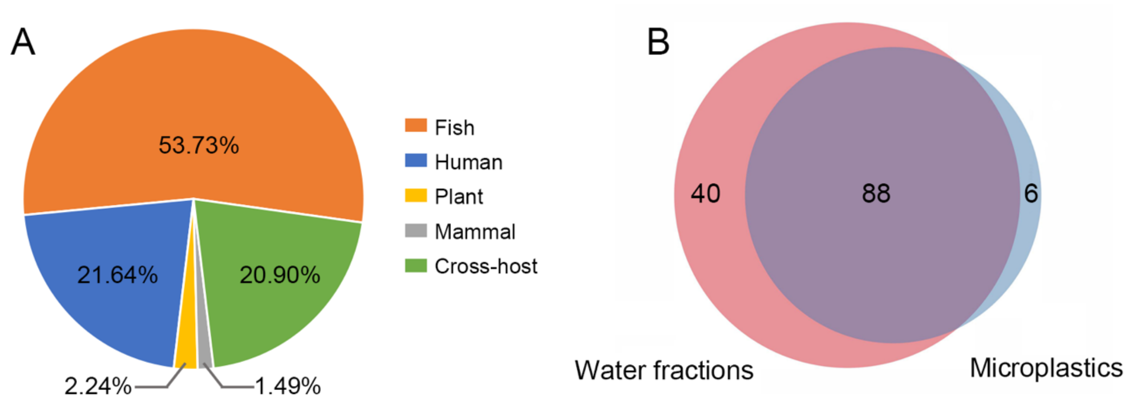


**Figure S2.** Overview of potential bacterial pathogens recovered from microplastics and water fractions. (A) Proportions of potential bacterial pathogens from different host sources on/in microplastics and water fractions. (B) Venn diagram showing the number of potentially pathogenic OTUs that are specific or overlapped on/in microplastics and water fractions.

**Table S1.** Summary of pathogens information in database of bacterial pathogens in aquatic environment.

| | Human | Fish | Mammal | Invertebrate | Plant | Cross-host | Total |
|---|---|---|---|---|---|---|---|
| Genus | 99 | 126 | 48 | 10 | 24 | 42 | 221 |
| Species | 334 | 569 | 135 | 17 | 76 | 114 | 1097 |
| Strains | 845 | 7146 | 358 | 23 | 118 | 580 | 9070 |

**Table S2**. Permutational multivariate analysis of variance (PERMANOVA) results testing the effect of sampling day and substrate on composition of pathogenic community based on Bray-Curtis dissimilarity.

| Factor | % Explained | F. Model | $R^2$ | *p* |
|---|---|---|---|---|
| Sampling day | 23.34 | 18.25 | 0.23 | 0.001 |
| Substrate | 19.56 | 9.18 | 0.20 | 0.001 |
| Sampling day × Substrate | 16.17 | 2.53 | 0.16 | 0.001 |
| Residuals | 40.92 | | 0.41 | |
| Total | 100 | | 1 | |

**Table S3.** Analysis of similarity (ANOSIM) based on Bray-Curtis dissimilarity for comparison of pathogenic community compositions of microplastics or water fractions between different sampling days.

| | R | *p* |
|---|---|---|
| Microplastics | **0.361** | 0.001 |
| Water fractions | **0.444** | 0.001 |

Data in bold indicate significant differences ($p < 0.05$).

**Table S4.** Analysis of similarity (ANOSIM) and permutational multivariate analysis of variance (PERMANOVA) based on Bray-Curtis dissimilarity for comparison of pathogenic bacterial community composition between microplastics and water fractions at each sampling day.

| Sampling day | ANOSIM | | PERMANOVA | |
|---|---|---|---|---|
| | R | *p* | $R^2$ | *p* |
| Day 15 | **0.341** | 0.001 | **0.166** | 0.001 |
| Day 30 | **0.767** | 0.001 | **0.273** | 0.001 |
| Day 60 | **0.357** | 0.001 | **0.170** | 0.001 |
| Day 90 | **0.464** | 0.001 | **0.174** | 0.001 |

Data in bold indicate significant differences ($p < 0.05$).
